# Novel 2-phenyl-4H-chromen derivatives: synthesis and anti-inflammatory activity evaluation *in vitro* and *in vivo*

**DOI:** 10.1080/14756366.2022.2124983

**Published:** 2022-09-21

**Authors:** Yun Xiao, Yaoyao Yan, Juncheng Du, Xiaoxiao Feng, Famin Zhang, Xu Han, Yong Hu, Xinhua Liu

**Affiliations:** a School of Pharmacy, Inflammation and Immune Mediated Diseases Laboratory of Anhui Province, Anhui Medical University, Hefei, P. R. China; b Anhui Academy of Agricultural Sciences, Agricultural Products Processing Institute, Hefei, P. R. China

**Keywords:** 2-Phenyl-4H-chromen-4-one derivatives, anti-inflammatory, TLR4/MAPK pathway

## Abstract

It is significant to design, synthesise and optimise flavonoid derivatives with better anti-inflammatory activity. This study aims to design and synthesise a series of novel 2-phenyl-4H-chromen-4-one compounds with anti-inflammatory; among them, compound **8** was discovered as the best one. And then, the effects of compound **8** on the TLR4/MAPK signalling pathway was carried out *in vivo*, the results indicated that compound **8** could downregulate NO, IL-6, and TNF-α expression, and suppress LPS-induced inflammation by inhibiting the TLR4/MAPK pathways. Furthermore, compound **8** reduced inflammation by a mouse model of LPS-induced inflammatory disease in *vivo*. The results suggest that compound **8** has the potential against inflammation through regulating TLR4/MAPK pathway and can be assessed further for drug development.

## Graphcal Abstract

**Figure:**
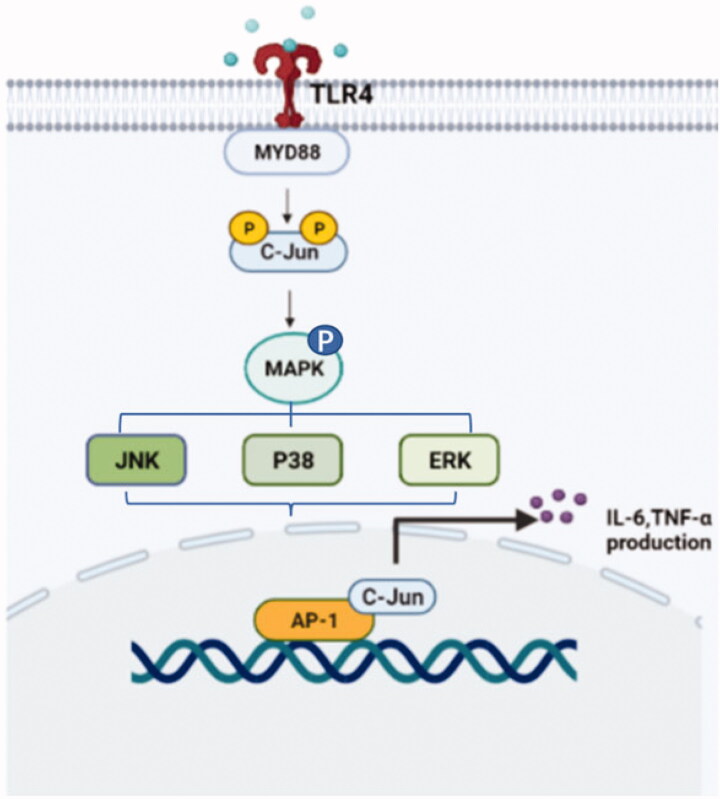

## Introduction

Inflammation is a common clinical-pathological process, which can occur in various tissues and organs of the body^1^. It is a defensive response and mainly characterised by redness, swelling, heat, and pain. Pro-inflammatory cytokines play an indispensable role in the defence response of disease^2^. However, some inflammatory diseases were caused by the uncontrolled and over secretion of pro-inflammatory cytokines^3–5^.

LPS is recognised by toll-like receptor 4 (TLR4), which is a major inducer of the immune response^6^. Stimulation of lipopolysaccharide (LPS) to TLR4 results in the activation of transcription factors, mainly mitogen-activated protein kinases (MAPKs)^7–9^. The activation of transcription factors leads to the production of many pro-inflammatory cytokines and chemokines, such as NO, tumour necrosis factor-α (TNF-α), and interleukin-6 (IL-6)^10^^,^^11^. Because inflammation is a major cause of various diseases including autoimmune diseases^12^, cancer^13^, inflammatory bowel disease^14–16^, drugs that inhibit the activation of the TLR4/MAPK signalling pathway are potential anti-inflammatory agents that prevent the development of many diseases.

Flavonoids, a group of polyphenolic compounds, are widely found in various plants, including fruits, vegetables, flowers, and tea^17^. Previous studies have successfully demonstrated that flavonoid derivatives had shown a broad range of pharmacological properties, especially anti-inflammation activity^18–22^. It is of great significance to design, synthesise and optimise flavonoid derivatives with better activity^23^. In this study, 12 new 2-phenyl-4H-chromen-4-one derivatives were synthesised. And then, the anti-inflammatory effect of the title compounds was evaluated *in vitro* and *in vivo*.

## Results

### Synthesis of compounds

**Scheme 1.**
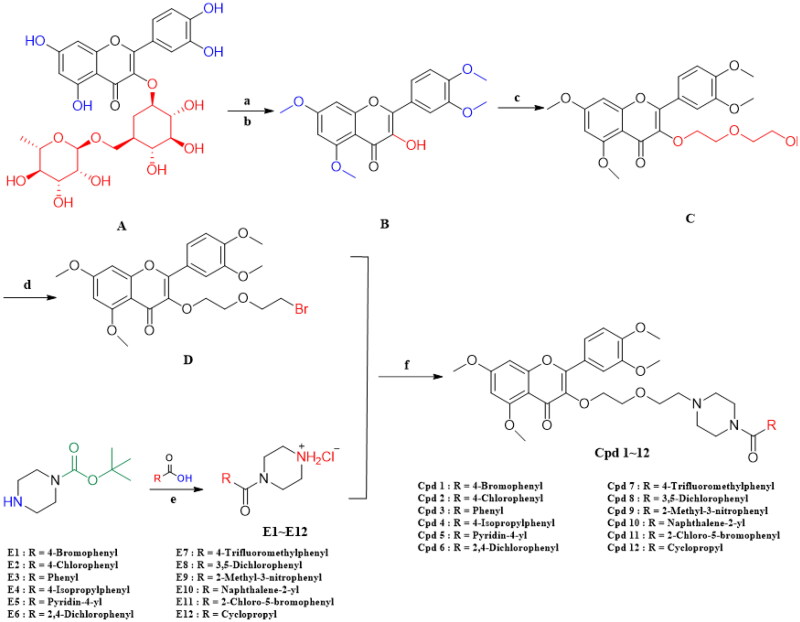
Reagents and conditions: (A) K_2_CO_3,_ (CH_3_)_2_SO_4_, acetone, 58 °C, reflux, 48 h. (B) concentrated hydrochloric acid, EtOH, room temperature, 2 h; (C) 2–(2-bromoethoxy)ethanol, KI, KOH, MeCN, 60 °C, 11 h; (D) (1) PBr_3_, MeCN, 50 °C, 1.5 h; (2) NaHCO_3_, H_2_O, cooled; (E) (1) EDCI, HOBT, Et_3_N, DCM, room temperature, 5 h; (2) EtOH, concentrated hydrochloric acid, 2 h; (F) (1) NaHCO_3_, H2O; (2) Cs_2_CO_3_, Pd(OAc)_2_, BINAP, PhMe, 2 h, 110 °C.

Rutin (**A**) was subjected to phenolic hydroxymethylation (a) followed by remove rhamnose (b) to obtain **B**, then the hydroxyl group was etherised with halohydrocarbons (c) to obtain intermediate **C**, the hydroxyl bromide of intermediate **C** (d) to obtain intermediate D, while the amino protected piperazine was condensed with acid amide, followed by deamination of the protecting group (e) to obtain another intermediate **E**, and intermediates **D** and **E** were nucleophilic substituted with ammonia and halohydrocarbons (f) to obtain the final product **F** (Scheme 1). Compounds are shown in Table 1.

**Table 1.** The structure of compounds **1–12**.

No.	R	No.	R	No.	R
**1**	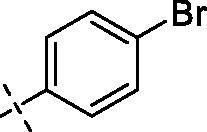	**5**	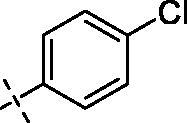	**9**	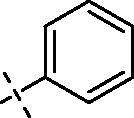
**2**	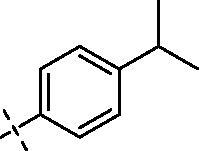	**6**	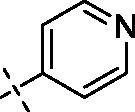	**10**	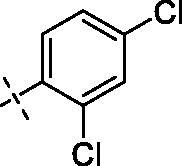
**3**	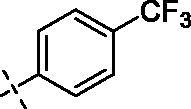	**7**	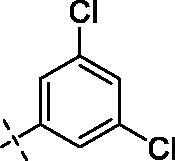	**11**	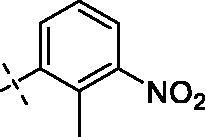
**4**	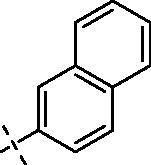	**8**	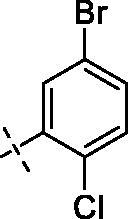	**12**	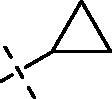

### Inhibitory activity against LPS-induced NO release

**Figure 1.**
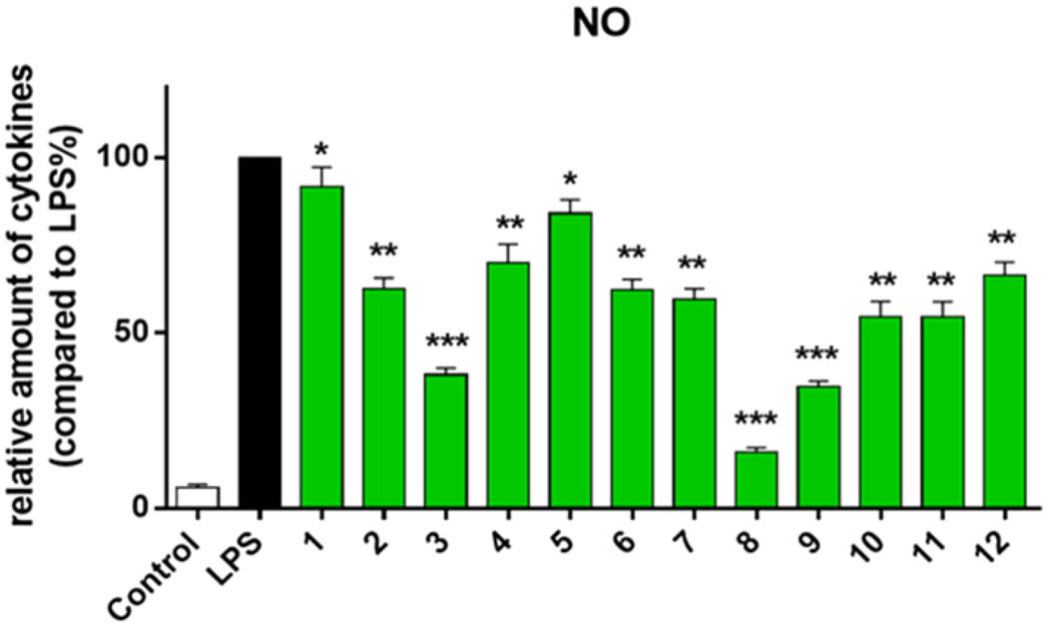
Inhibiting of NO production by all compounds **1–12**. RAW264.7 cells were pre-treated with compounds **1–12** (20 μM) for 1 h, then incubated with LPS for another 24 h. ***p < 0.001; **p < 0.01; *p < 0.05 vs. LPS group.

In order to evaluate the anti-inflammatory activity of compounds, the ability of all compounds to inhibit NO over-expression by LPS in RAW264.7 cells were measured. RAW264.7 cells were pre-incubated with compound (20 μM) for 1 h and then treated with LPS (0.5 μg/ml) for 24 h. The concentration of NO in the medium was detected by Griess Reagent Assay. The results indicated that the LPS-induced NO production could be reduced by most of the synthetic compounds at 20 μM (Figure 1). Among them, compound **8** showed strong inhibitory activity.

### Assessment of toxicity

**Figure 2.**
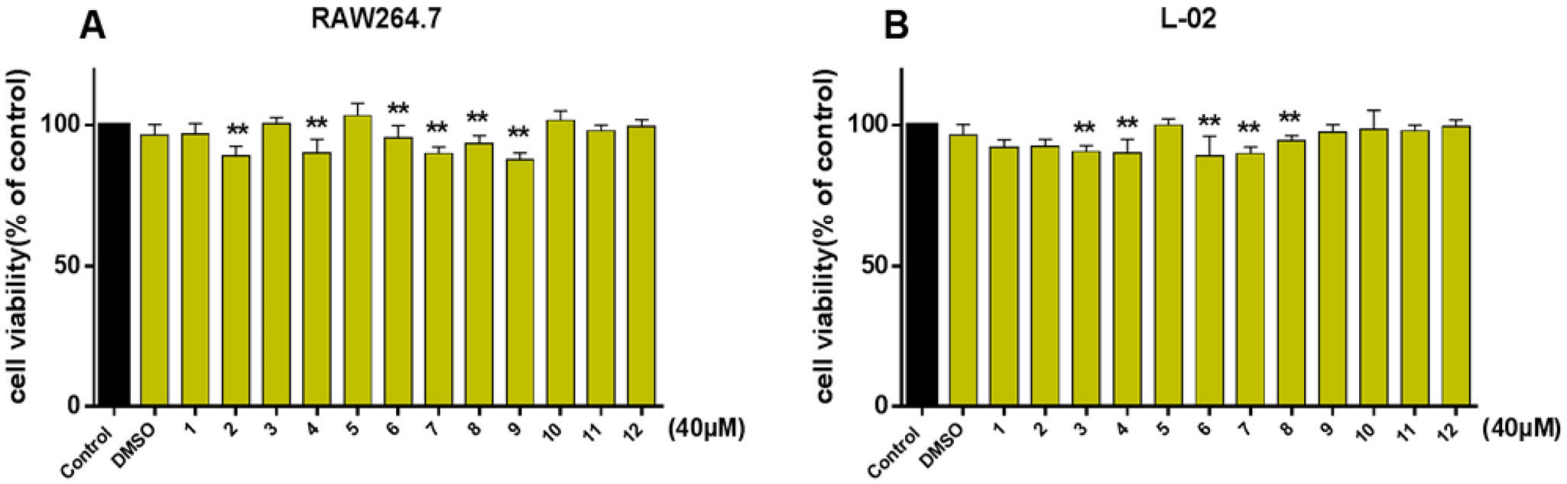
The cytotoxic evaluation in cells. The cell viability was evaluated by the MTT assay. (A) Effects of compounds **1–12** on RAW264.7 cells. (B) Effects of compounds **1–12** on L-02 cells. **p < 0.05 compare with the control group.

We then evaluated the cytotoxicity of compounds using the MTT assay on the RAW264.7 and L-02 cells. The results showed that all compounds had no obvious toxicity at 40 μM, and the cell viability had no significant effect compared with the normal group (Figure 2).

### Inhibiting of NO, IL-6, and TNF-α production by compound 8

**Figure 3.**
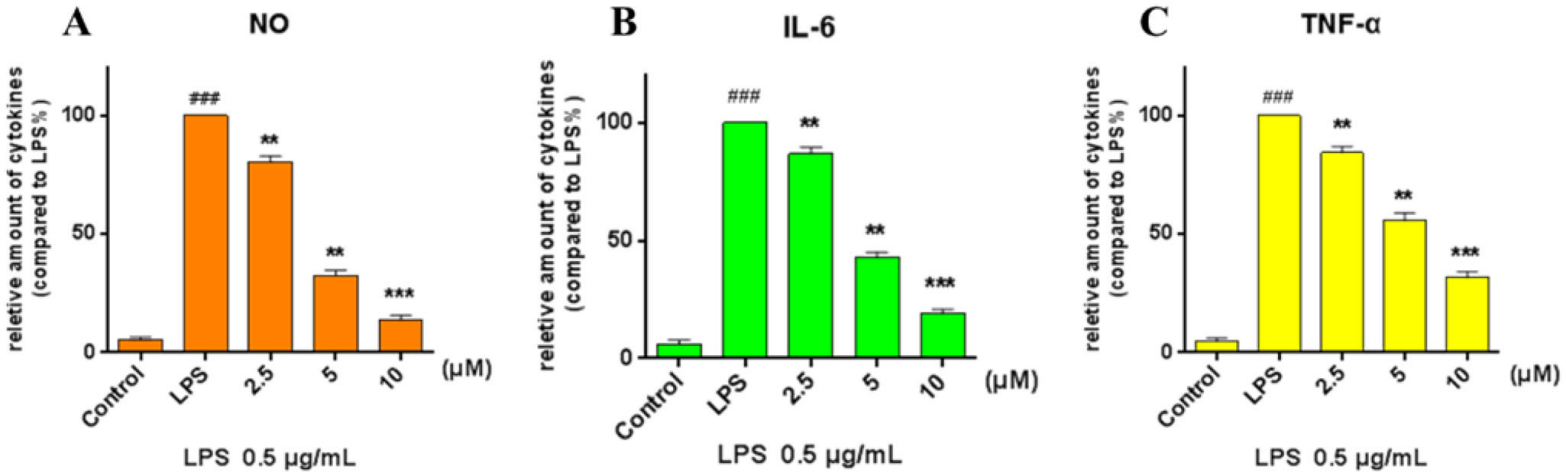
Inhibiting of the cytokine production. RAW264.7 cells were pre-treated with compound **8** at concentrations of 10, 5, and 2.5 μM for 1 h, incubated with LPS (0.5 μg/ml) for 24 h, (A) NO production was measured using Griess Reagent assay. The levels of (B) IL-6 and (C) TNF-α in the culture medium were measured by ELISA. ***p < 0.001; **p < 0.01; *p < 0.05 vs. LPS group.

IL-6 and TNF-α have been shown to exert regulating effects on the inflammation^24^^,^^25^. So, compound **8** with better NO inhibition activity was selected to tested the ability to inhibit the production of IL-6 and TNF-α. RAW264.7 were pre-treated with compound **8** in different concentrations (10, 5, and 2.5 μM) for 1 h and then treated with LPS (0.5 μg/ml) for 24 h. The cell supernatant was collected, the level of NO was measured by Griess Reagent Assay, and the level of IL-6 and TNF-α was detected by ELISA kits. As shown in Figure 3, compound **8** significantly decreased the release of NO, IL-6, and TNF-α in a concentration-dependent manner.

### Effect of compound 8 on TLR4/MAPK signalling pathway

**Figure 4.**
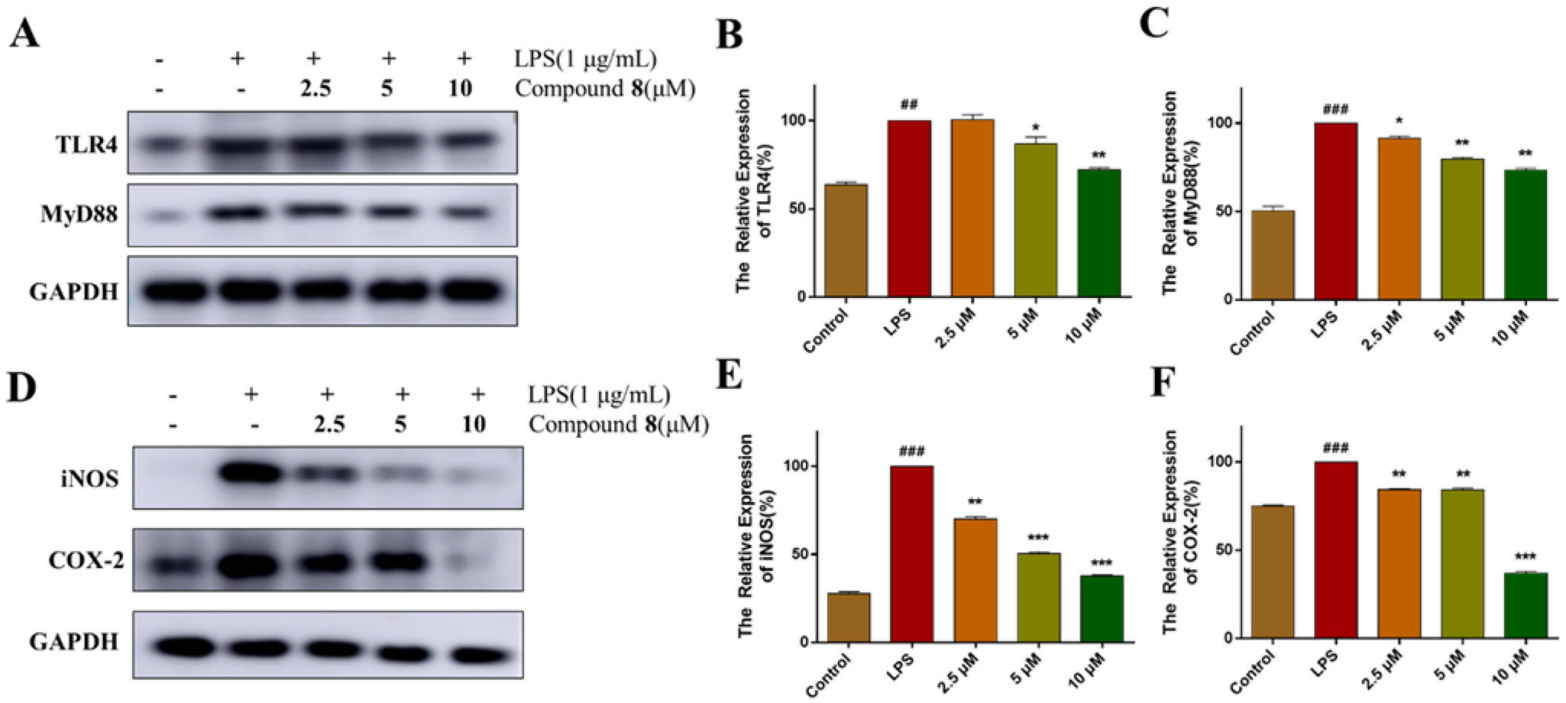
Compound **8** suppressed the activation of TLR4 signalling. (A–C) The expression of TLR4 and MyD88 were analysed using western blotting. (D–F) The expression of iNOS and COX-2 were analysed using western blotting. ^###^*p* < 0.001 vs. control group; ***p < 0.001; **p < 0.01; *p < 0.05 vs. LPS group.

TLRs are a significant family in the innate immune system that activate downstream inflammatory signalling pathways in response to exogenous stimulation^26^. It is reported that LPS-stimulation induced the activation of TLR4, leading to the expression of MyD88^27^. As shown in Figure 4(A–C), RAW264.7 cells were stimulated with LPS, the expression of TLR4 and downstream signal MyD88 were increased, which was reversed after being treated with compound **8** in a dose-dependent manner. Activation of TLR4 pathways leads to high expression of iNOS and COX-2 protein, which is related to the modulation expression of NO. Similarly, the expression of iNOS and COX-2 were decreased by treatment with compound **8** (Figure 4(D–F)). The results indicated that compound **8** suppressed LPS-induced inflammatory response in RAW264.7 cells.

**Figure 5.**
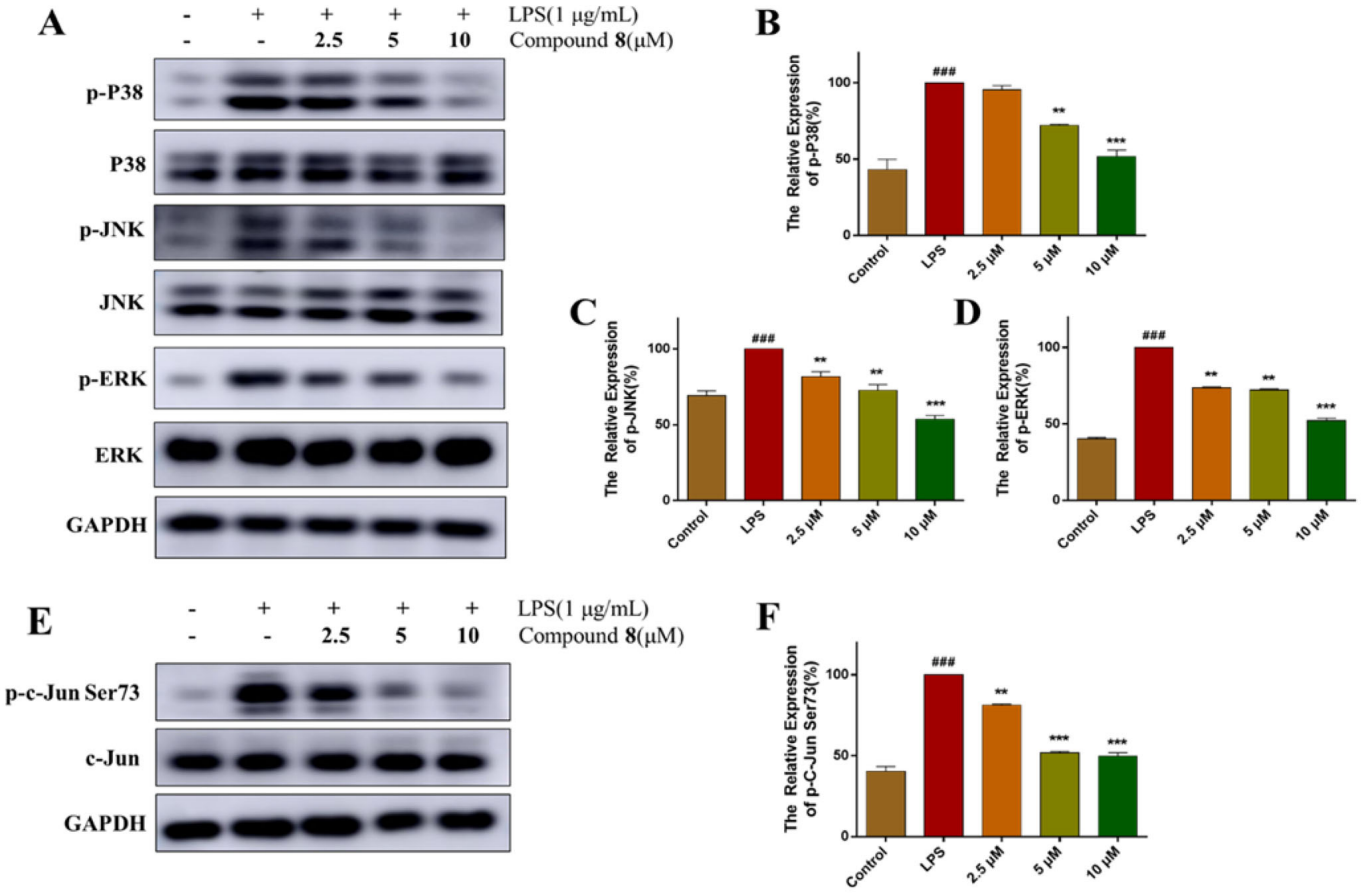
Compound **8** suppressed the activation of MAPK signalling. (A–D) The expression of phosphorylation of MAPK were analysed using western blotting. (E,F) The expression of phosphorylation of c-Jun were analysed using western blotting. ^###^*p* < 0.001 vs. control group; ***p < 0.001; **p < 0.01; *p < 0.05 vs. LPS group.

The activation of TLR4 results in the activation of signalling pathway including MAPK pathway, then the transcription factors AP-1 translocate into the nucleus to phosphorylate c-Jun at Ser73 and bind to target promoters turn on transcriptions of inflammatory genes including the production of NO, TNF-α, IL-6, and other inflammatory mediators. So, we analysed the levels of phosphorylation of MAPK and c-Jun using western blotting. As shown in the results, P38, JNK, and ERK were phosphorylated by LPS and phosphorylation levels were reversed after treatment with compound **8** (Figure 5(A–D)). Similarly, the phosphorylation of c-Jun Ser73 was activated by LPS and compound **8** decreased the phosphorylation level in a dose-dependent manner (Figure 5(E,F)).

### In vivo activity of compound 8

**Figure 6.**
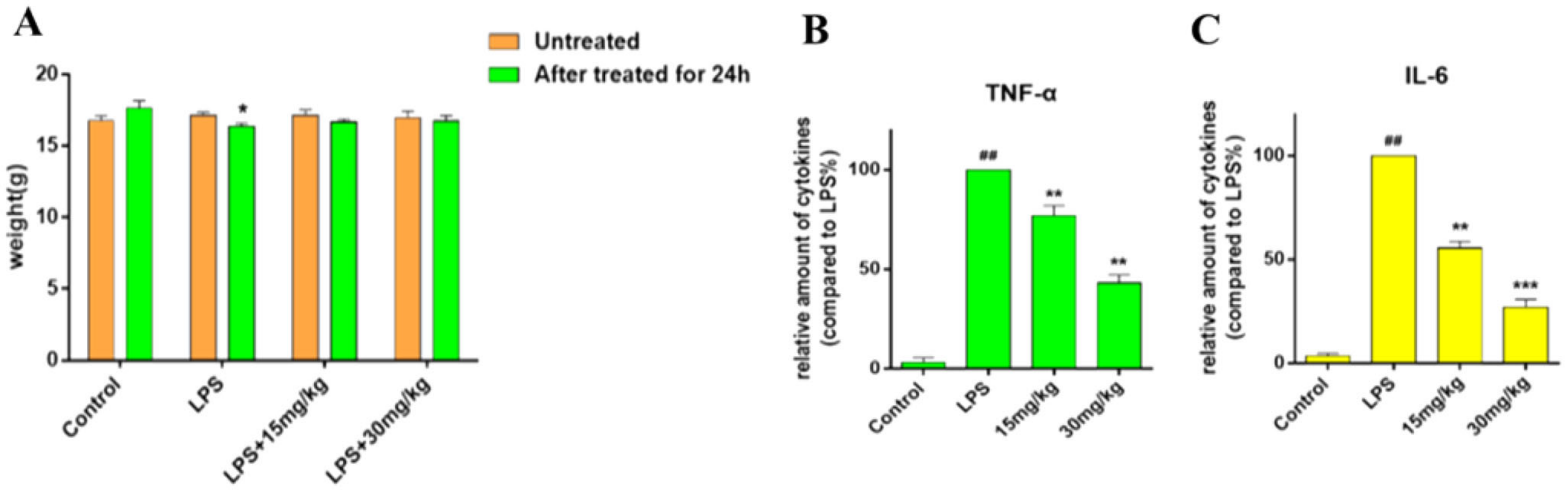
Compound **8** protected LPS-induced inflammatory disease. C57BL/6 mice were treated by intraperitoneal injection with compound **8** (15 and 30 mg/kg), and after 30 min, were administrated with 5 mg/kg LPS by tracheal instillation. (A) Body weight changes. (B) The level of IL-6 in serum. (C) The level of TNF-α in serum. ^###^*p* < 0.001 compared with control group; **p < 0.01; ***p < 0.001 compare with LPS group.

In order to evaluate the anti-inflammatory activity *in vivo* of compound **8**, LPS-induced inflammatory disease model was used. C57BL/6 mice were treated by intraperitoneal injection with compound **8** (15 and 30 mg/kg), and after 30 min were administrated with 5 mg/kg LPS by tracheal instillation. As shown in Figure 6(B,C), the levels of IL-6 and TNF-α in serum in the LPS group were significantly increased compared to the control group, and the administration of compound **8** effectively prevented the increase. Besides, body weight was reduced due to LPS, however, compound **8** seemed to have a significant effect on the weight loss induced by LPS (Figure 6(A)).

## Conclusion

To discover highly effective anti-inflammatory compounds, based on flavonoid moiety, twelve 2-phenyl-4H-chromen-4-one derivatives were designed and synthesised. The initial evaluation results showed that most compounds had favourable NO inhibitory activity and low toxicity in RAW264.7 cells. More importantly, one compound suppressed the release of pro-inflammatory cytokines through the inhibiting of the TLR4/MAPK signalling pathways in RAW264.7 cells. The more study *in vivo* demonstrated that this compound had low toxicity and could significantly decrease the level of IL-6 and TNF-α in serum. In summary, the title compound has potential against inflammation and can be assessed further for drug development.

## Materials and methods

### Chemistry

#### Structure characterisation

^1^H and ^13^C NMR spectral data were recorded with a Bruker spectrometer using tetramethylsilane (TMS) as internal standard (IS). ^1^H NMR and ^13^C spectra were recorded at 600 and 151 MHz, deuterium chloroform was used as the reference solvent.

#### Synthetic of 2–(3,4-dimethoxyphenyl)-3-hydroxy-5,7-dimethoxy-4H-chromen-4-one (B)

Rutin (6.10 g, 10 mmol) and K_2_CO_3_ (27.64 g, 300 mmol) were added to a 500 ml round-bottomed flask in turn, and acetone was added to dissolve, and after stirring for 1 h under reflux. Then dimethyl sulphate (14.28 ml, 300 mmol) was slowly added dropwise to the reaction system and stirred at room temperature for 48 h, while the reaction was followed by TLC (dichloromethane: methanol = 12.5: 1, *V/V*). After the reaction stopped, filter the precipitate, wash the filter residue with methanol, combine the filtrates, and concentrate under reduced pressure. The concentrate was then dissolved in 100 ml of water and the temperature was raised to the temperature suitable for reflux. After the solution became clear, 20 ml of concentrated hydrochloric acid was slowly added under reflux. Then, the yellow solid was precipitated, and the reaction was continued for 2 h. The reaction system was cooled and filtered to obtain a crude product. Compound **B** was directly used in the next step without purification (yield: 70%).

#### Synthetic of 2–(3,4-dimethoxyphenyl)-3–(2–(2-hydroxyethoxy)ethoxy)-5,7-dimethoxy-4H-chromen-4-one (C)

To the solution of **B** (2 g, 5.58 mmol) in 100 ml of MeCN, potassium hydroxyde (0.627 g, 11.16 mmol) was added and the resultant mixture was heated to 60 °C. After 2 h, potassium iodide (0.926 g, 5.58 mmol) and a solution of 2-(2-bromoethoxy)ethanol (1.88 g, 11.16 mmol) in 3.0 ml of MeCN were added and the reaction mixture was stirred for additional 10 h at 60 °C. Then, the mixture was diluted with water, extracted with ethyl acetate, and the organic layers dried over anhydrous sodium sulphate, filtered, and concentrated. Purified by silica gel column chromatography (40% ethyl acetate in n-hexane solution), the pale yellow oil **C** was obtained with a yield of 83%.

#### Synthetic of 3–(2-(2-bromoethoxy)ethoxy)-2–(3,4-dimethoxyphenyl)-5,7-dimethoxy-4H-chromen-4-one (D)

Compound **C** (2 g, 4.48 mmol) and anhydrous acetonitrile were dissolved in a 100 ml round bottom flask and heated to reflux, and PBr_3_ (0.37 g, 1.5 mmol) was added dropwise to the mixture. The mixed system was heated continuously for 1.5 h. The reaction mixture was then poured onto ice and the resulting mixture was neutralised with 15% NaHCO_3_.The obtained mixture was extracted into toluene, the organic phase was dried with Na_2_SO_4_, and the solvent was evaporated to obtain **D** with a yield of 73%.

#### Synthetic of 4–(4-bromobenzoyl)piperazin-1-ium chloride (E1)

4-Bromobenzoic acid (10 mmol), EDCI (2.88 g, 15 mmol), HOBT (2.03 g, 15 mmol) were sequentially added to a 100 ml round bottom flask, and dissolved in dichloromethane. After the reaction system was stirred at room temperature for 0.5 h, 1-tert-butoxycarbonylpiperazine (2.24 g, 12 mmol) was added, and the reaction was continued for 3 h at room temperature. The reaction was followed by TLC (petroleum ether: ethyl acetate = 2: 1, *V/V*). After stopping the reaction, the solution was dispersed with 200 ml of water, and extracted three times with dichloromethane. The organic layers were combined, and the organic layers were washed twice with water and saturated NaCl. The resulting organic layer was dried over anhydrous Na_2_SO_4_ and concentrated under reduced pressure. After purification by column chromatography (petroleum ether: ethyl acetate = 2:1, *V/V*), the obtained product was dissolved in 10 ml of absolute ethanol. To the mixture was added 5 ml of concentrated hydrochloric acid and reacted at room temperature for 2 h. After concentration under reduced pressure, **E1** was obtained in 95% yield.

#### General procedure for the synthesis of compounds 1–12

After neutralising **E1** with saturated NaHCO_3_ solution, it was extracted with DCM and the organic layer was concentrated. Concentrate (0.4 g, 3.12 mmol), **D** (1.9 g, 3.74 mmol), Pd(OAc)_2_ (0.035 g, 0.156 mmol), BINAP (5.83 g, 9.36 mmol), CS_2_CO_3_ (3.05 g, 9.36 mmol) and toluene (10 ml) was stirred at 110 °C for 2 h in a sealed tube. The mixture was diluted (EtOAc), filtered, and concentrated. After purification, **1** was obtained. Compounds **2 ∼ 12** were prepared as for compound **1**.

#### 3–(2-(2–(4-(4-Bromobenzoyl)piperazin-1-yl)ethoxy)ethoxy)-2–(3,4 dimethoxyphenyl)-5,7-dimethoxy-4H-chromen-4-one (1)

Yield: 78%; yellow oil; ^1^H NMR (600 MHz, CDCl3) *δ* 7.77 (d, *J* = 1.8 Hz, 1H), 7.72 (dd, *J* = 8.5, 1.9 Hz, 1H), 7.53 (d, *J* = 8.3 Hz, 2H), 7.26 (d, *J* = 8.3 Hz, 2H), 6.95 (d, *J* = 8.6 Hz, 1H), 6.50 (d, *J* = 2.1 Hz, 1H), 6.34 (d, *J* = 2.0 Hz, 1H), 4.25–4.21 (m, 2H) (Ar–O–CH_2_–), 3.98–3.93 (m, 9H) (–OCH_3_)_3_, 3.90 (s, 3H) (–OCH_3_), 3.77–3.67 (m, 4H) (–CH_2_–O–CH_2_), 3.54 (t, *J* = 5.5 Hz, 2H) (–CH_2_–N–C = O), 3.36 (s, 2H) (–CH_2_–N–C = O), 2.51 (s, 4H) (N– (CH_2_)_2_, 2.39 (s, 2H) (N–CH_2_). ^3^C NMR (151 MHz, CDCl_3_) *δ* 178.36 (Ar–CO–), 169.11 (–CO–N), 164.19, 160.00, 158.17, 149.99, 149.31, 135.96, 134.25, 131.20, 129.61 (2 C), 124.33, 121.40 (2 C), 111.79, 111.60, 107.33, 96.11, 92.57 (Ar–O–CH–), 70.21, 69.98, 65.66, 56.91 (2 C), 55.97 (2 C), 56.19 (2 C), 48.97, 43.66 (2 C). ESI Calcd for [M + H] C_34_H_37_N_2_O_9_Br^+^ 697.1755, found 697.1758.

#### 3–(2-(2–(4-(4-Chlorobenzoyl)piperazin-1-yl)ethoxy)ethoxy)-2–(3,4-dimethoxyphenyl)-5,7-dimethoxy-4H-chromen-4-one (2)

Yield: 81%; yellow oil; ^1^H NMR (600 MHz, CDCl_3_) *δ* 7.76 (d, *J* = 1.7 Hz, 1H), 7.71 (dd, *J* = 8.5, 1.9 Hz, 1H), 7.36 (d, *J* = 8.4 Hz, 2H), 7.32 (d, *J* = 8.4 Hz, 2H), 6.94 (d, *J* = 8.6 Hz, 1H), 6.49 (d, *J* = 2.0 Hz, 1H), 6.33 (d, *J* = 2.0 Hz, 1H), 4.25–4.20 (m, 2H) (Ar–O–CH_2_–), 3.97–3.92 (m, 9H) (–OCH_3_)_3_, 3.89 (s, 3H) (–OCH_3_), 3.76–3.65 (m, 4H) (–CH_2_–O–CH_2_), 3.53 (t, *J* = 5.5 Hz, 2H)( –CH_2_–N–C = O), 3.35 (s, 2H) (–CH_2_–N–C = O), 2.50 (t, *J* = 5.1 Hz, 4H) (N–(CH_2_)_2_, 2.38 (s, 2H) (N–CH_2_); ^13^C NMR (151 MHz, CDCl_3_) *δ* 173.86 (Ar–CO–), 169.08 (–CO–N), 163.91, 160.96, 158.72, 152.66, 150.79, 148.52, 139.96, 135.67, 134.12, 128.67 (2 C), 128.57 (2 C), 123.40, 121.70, 111.88, 110.65, 109.33, 95.75, 92.43 (Ar–O–CH–), 71.02, 70.38, 68.54, 57.57, 56.33, 56.10, 55.92, 55.72, 53.69, 53.22, 47.60, 42.07. ESI Calcd for [M + H] C_34_H_37_N_2_O_9_Cl^+^ 653.2261, found 653.2261.

#### 3–(2-(2–(4-Benzoylpiperazin-1-yl)ethoxy)ethoxy)-2–(3,4-dimethoxyphenyl)-5,7-dimethoxy-4H-chromen-4-one (3)

Yield: 79%; yellow oil; ^1^H NMR (600 MHz, CDCl_3_) *δ* 7.75 (s, 1H), 7.72 (d, *J* = 8.5 Hz, 1H), 7.38 (s, 5H), 6.95 (d, *J* = 8.5 Hz, 1H), 6.50 (d, *J* = 1.5 Hz, 1H), 6.34 (d, *J* = 1.3 Hz, 1H), 4.22 (s, 2H) (Ar–O–CH_2_–), 3.95 (d, *J* = 10.1 Hz, 9H) (–OCH_3_)_3_, 3.90 (s, 3H) (–OCH_3_), 3.73 (s, 4H) (–CH_2_–O–CH_2_), 3.58 (s, 2H) (–CH_2_–N–C = O), 3.43 (s, 2H) (–CH_2_–N–C = O), 2.49 (d, *J* = 63.8 Hz, 6H) (N–(CH_2_)_2_ and (N–CH_2_). ^13^C NMR (151 MHz, CDCl_3_) *δ* 175.99 (Ar–CO–), 169.17, 164.02, 160.91, 158.99, 157.35, 150.00, 149.32, 136.25, 135.60, 129.44, 128.13 (2 C), 127.61 (2 C), 122.45, 121.21, 111.85, 111.63, 107.55, 95.92, 92.40 (Ar–O–CH–), 70.69, 70.30, 66.21, 57.02 (2 C), 56.10 (2 C), 56.19 (2 C), 48.24, 43.89. ESI Calcd for [M + H] C_34_H_38_N_2_O_9_^+^ 619.265, found 619.2656.

#### 2–(3,4-Dimethoxyphenyl)-3–(2-(2–(4-(4-isopropylbenzoyl)piperazin-1-yl)ethoxy)ethoxy)-5,7-dimethoxy-4H-chromen-4-one (4)

Yield: 67%; yellow oil; ^1^H NMR (600 MHz, CDCl_3_) *δ* 7.77 (d, *J* = 1.7 Hz, 1H), 7.72 (dd, *J* = 8.5, 1.9 Hz, 1H), 7.30 (d, *J* = 8.1 Hz, 2H), 7.23 (d, *J* = 8.0 Hz, 2H), 6.95 (d, *J* = 8.6 Hz, 1H), 6.50 (d, *J* = 2.0 Hz, 1H), 6.34 (d, *J* = 1.9 Hz, 1H), 4.26–4.21 (m, 2H), 3.97–3.93 (m, 9H) (–OCH_3_)_3_, 3.90 (s, 3H) (–OCH_3_), 3.79–3.68 (m, 4H), 3.53 (t, *J* = 5.4 Hz, 2H), 3.41 (s, 2H), 2.91 (dt, *J* = 13.7, 6.9 Hz, 1H) CH (CH_3_)_2_, 2.50 (s, 4H), 2.38 (s, 2H), 1.24 (d, *J* = 6.9 Hz, 6H) CH (CH_3_)_2_. ^13^C NMR (151 MHz, CDCl_3_) *δ* 175.01 (Ar–CO–), 168.68 (–CO–N), 163.99, 160.00, 158.55, 150.12, 150.00, 149.66, 149.11, 135.99, 132.65, 127.10 (2 C), 126.22 (2 C), 121.85, 121.33, 111.78, 111.55, 107.67, 96.30, 92.81 (Ar–O–CH–), 70.58, 69.97, 66.01, 56.95 (2 C), 56.34 (2 C), 55.92, 47.89, 43.29 (2 C), 33.39, 24.11 (2 C). ESI Calcd for [M + H]^+^ C_37_H_44_N_2_O_9_^+^ 661.312, found 661.3130.

#### 2–(3,4-Dimethoxyphenyl)-3–(2-(2–(4-isonicotinoylpiperazin-1-yl)ethoxy)ethoxy)-5,7-dimethoxy-4H-chromen-4-one (5)

Yield: 65%; yellow oil; ^1^H NMR (600 MHz, CDCl_3_) *δ* 8.66 (d, *J* = 5.6 Hz, 2H), 7.76 (d, *J* = 1.3 Hz, 1H), 7.70 (dd, *J* = 8.5, 1.7 Hz, 1H), 7.25 (dd, *J* = 4.5, 1.2 Hz, 2H), 6.94 (d, *J* = 8.6 Hz, 1H), 6.49 (d, *J* = 1.8 Hz, 1H), 6.33 (d, *J* = 1.7 Hz, 1H), 4.24–4.20 (m, 2H), 3.94 (d, *J* = 9.8 Hz, 9H) (–OCH_3_)_3_, 3.89 (s, 3H) (–OCH_3_), 3.73 (s, 4H), 3.54 (t, *J* = 5.3 Hz, 2H), 3.30 (s, 2H), 2.51 (s, 4H), 2.39 (s, 2H); ^13^C NMR (151 MHz, CDCl_3_) *δ* 173.85 (Ar–CO–), 167.48 (–CO–N), 163.93, 160.98, 158.73, 152.66, 150.83, 150.20 (2 C), 148.56, 143.44, 139.96, 123.43, 121.70, 121.19 (2 C), 111.95, 110.71, 109.35, 95.78, 92.46 (Ar–O–CH–), 71.01, 70.39, 68.55, 57.53, 56.33, 56.13, 55.93, 55.72, 53.63, 53.01, 47.27, 41.88. ESI Calcd for [M + H]^+^ C_33_H_37_N_3_O_9_^+^ 620.2603, found 620.2599.

#### 3–(2-(2–(4-(2,4-Dichlorobenzoyl)piperazin-1-yl)ethoxy)ethoxy)-2–(3,4-dimethoxyphenyl)-5,7-dimethoxy-4H-chromen-4-one (6)

Yield: 77%; yellow oil; ^1^H NMR (600 MHz, CDCl_3_) *δ* 7.77 (s, 1H), 7.72 (d, *J* = 8.5 Hz, 1H), 7.41 (s, 1H), 7.29 (d, *J* = 8.2 Hz, 1H), 7.20 (d, *J* = 8.2 Hz, 1H), 6.95 (d, *J* = 8.5 Hz, 1H), 6.50 (d, *J* = 1.4 Hz, 1H), 6.34 (d, *J* = 1.2 Hz, 1H), 4.25–4.21 (m, 2H), 3.95 (d, *J* = 6.6 Hz, 9H) (–OCH_3_)_3_, 3.90 (s, 3H) (–OCH_3_), 3.74 (dd, *J* = 11.4, 7.1 Hz, 4H), 3.53 (t, *J* = 5.3 Hz, 2H), 3.19 (dt, *J* = 16.5, 11.7 Hz, 2H), 2.50 (s, 4H), 2.40 (d, *J* = 66.6 Hz, 2H);^13^C NMR (151 MHz, CDCl_3_) *δ* 173.85 (Ar–CO–), 165.74 (–CO–N), 163.90, 160.99, 158.73, 152.62, 150.81, 148.54, 139.98, 135.42, 134.32, 131.31, 129.52, 128.72, 127.53, 123.44, 121.70, 111.92, 110.67, 109.37, 95.76, 92.44 (Ar–O–CH–), 71.02, 70.40, 68.63, 57.54, 56.34, 56.12, 55.93, 55.72, 53.56, 52.98, 46.60, 41.58. ESI Calcd for [M + H]^+^ C_34_H_36_N_2_O_9_Cl_2_^+^ 687.1871, found 687.1877.

#### 2–(3,4-Dimethoxyphenyl)-5,7-dimethoxy-3–(2-(2–(4-(4-(trifluoromethyl)benzoyl)piperazin-1-yl)ethoxy)ethoxy)-4H-chromen-4-one (7)

Yield: 68%; yellow oil; ^1^H NMR (600 MHz, CDCl_3_) *δ* 7.77 (s, 1H), 7.72 (dd, *J* = 8.5, 1.7 Hz, 1H), 7.66 (d, *J* = 8.0 Hz, 2H), 7.49 (d, *J* = 7.9 Hz, 2H), 6.95 (d, *J* = 8.6 Hz, 1H), 6.50 (d, *J* = 1.9 Hz, 1H), 6.34 (d, *J* = 1.8 Hz, 1H), 4.25–4.20 (m, 2H), 3.95 (d, *J* = 9.8 Hz, 9H) (–OCH_3_)_3_, 3.90 (s, 3H) (–OCH_3_), 3.74 (d, *J* = 4.4 Hz, 4H), 3.56 (s, 2H), 3.34 (s, 2H), 2.53 (s, 4H), 2.41 (s, 2H). ^13^C NMR (151 MHz, CDCl_3_) *δ* 176.50 (Ar–CO–), 168.44 (–CO–N), 163.95, 160.88, 159.82, 149.81, 149.14, 138.66, 135.93, 127.39, 125.24 (2 C), 123.95, 122.20, 121.00, 110.89, 110.65, 107.00, 95.90, 92.40 (Ar–O–CH–), 70.55, 70.00, 65.99, 56.88 (2 C), 56.10 (2 C), 56.00 (2 C), 48.87, 43.87 (2 C). ESI Calcd for [M + H]^+^ C_35_H_37_N_2_O_9_F_3_^+^ 687.2524, found 687.2525.

#### 3–(2-(2–(4-(3,5-Dichlorobenzoyl)piperazin-1-yl)ethoxy)ethoxy)-2–(3,4-dimethoxyphenyl)-5,7-dimethoxy-4H-chromen-4-one (8)

Yield: 75%; yellow oil; ^1^H NMR (600 MHz, CDCl_3_) *δ* 7.77 (d, *J* = 1.6 Hz, 1H), 7.71 (dd, *J* = 8.5, 1.8 Hz, 1H), 7.39 (s, 1H), 7.24 (d, *J* = 1.7 Hz, 2H), 6.94 (d, *J* = 8.6 Hz, 1H), 6.50 (d, *J* = 2.0 Hz, 1H), 6.33 (d, *J* = 1.9 Hz, 1H), 4.25–4.21 (m, 2H), 3.95 (d, *J* = 8.8 Hz, 9H) (–OCH_3_)_3_, 3.89 (s, 3H) (–OCH_3_), 3.72 (dd, *J* = 12.6, 8.3 Hz, 4H), 3.54 (t, *J* = 5.4 Hz, 2H), 3.34 (s, 2H), 2.50 (d, *J* = 5.1 Hz, 4H), 2.39 (s, 2H); ^13^C NMR (151 MHz, CDCl_3_) *δ* 173.85 (Ar–CO–), 167.06 (–CO–N), 163.90, 160.99, 158.73, 152.62, 150.81, 148.54, 139.97, 138.64, 135.32 (2 C), 129.64, 125.46 (2 C), 123.45, 121.68, 111.95, 110.67, 109.37, 95.75, 92.43 (Ar-O-CH-), 71.02, 70.40, 68.56, 57.54, 56.34, 56.11, 55.92, 55.72, 53.61, 53.00, 47.53, 42.12. ESI Calcd for [M + H]^+^ C_34_H_36_N_2_O_9_Cl_2_^+^ 687.1871, found 687.1875.

#### 2–(3,4-Dimethoxyphenyl)-5,7-dimethoxy-3–(2-(2–(4-(2-methyl-3-nitrobenzoyl)piperazin-1-yl)ethoxy)ethoxy)-4H-chromen-4-one (9)

Yield: 69%; yellow oil; ^1^H NMR (600 MHz, CDCl_3_) *δ* 7.87 (dd, *J* = 6.2, 3.1 Hz, 1H), 7.76 (d, *J* = 1.8 Hz, 1H), 7.70 (dd, *J* = 8.5, 1.9 Hz, 1H), 7.40–7.35 (m, 2H), 6.94 (d, *J* = 8.6 Hz, 1H), 6.50 (d, *J* = 2.1 Hz, 1H), 6.33 (d, *J* = 2.0 Hz, 1H), 4.24–4.20 (m, 2H), 3.94 (d, *J* = 8.1 Hz, 9H) (–OCH_3_)_3_, 3.89 (s, 3H) (–OCH_3_), 3.78 (s, 2H), 3.75–3.71 (m, 2H), 3.54 (t, *J* = 5.5 Hz, 2H), 3.16 (s, 2H), 2.55–2.48 (m, 4H), 2.44 (s, 3H) (Ar–CH_3_), 2.36 (dd, *J* = 13.9, 9.8 Hz, 2H). ^13^C NMR (151 MHz, CDCl_3_) *δ* 174.33 (Ar–CO–), 166.65 (–CO–N), 164.30, 160.95, 159.86, 158.45, 150.03, 149.39, 137.60, 135.88, 133.33, 131.60, 126.51, 125.20, 122.80, 121.93, 111.55, 111.31, 107.70, 96.22, 92.33 (Ar–O–CH–), 70.45, 69.99, 65.64, 56.79 (2 C), 55.90 (2 C), 55.98 (2 C), 48.22, 43.10 (2 C), 13.69. ESI Calcd for [M + H]^+^ C_35_H_39_N_3_O_11_^+^ 678.2657, found 678.2661.

#### 3–(2-(2–(4-(2-Naphthoyl)piperazin-1-yl)ethoxy)ethoxy)-2–(3,4 dimethoxyphenyl)-5,7-dimethoxy-4H-chromen-4-one (10)

Yield: 75%; yellow oil; ^1^H NMR (600 MHz, CDCl_3_) *δ* 7.85 (dd, *J* = 16.7, 8.4 Hz, 4H), 7.76 (s, 1H), 7.71 (d, *J* = 8.5 Hz, 1H), 7.54–7.48 (m, 2H), 7.46 (d, *J* = 8.4 Hz, 1H), 6.93 (d, *J* = 8.5 Hz, 1H), 6.48 (s, 1H), 6.32 (s, 1H), 4.25–4.20 (m, 2H), 3.93 (d, *J* = 18.6 Hz, 9H) (–OCH_3_)_3_, 3.88 (s, 3H) (–OCH_3_), 3.79 (s, 2H), 3.75–3.71 (m, 2H), 3.54 (t, *J* = 5.5 Hz, 2H), 3.44 (s, 2H), 2.51 (t, *J* = 5.6 Hz, 4H), 2.40 (s, 2H); ^13^C NMR (151 MHz, CDCl_3_) *δ* 173.86 (Ar–CO–), 170.18 (–CO–N), 163.88, 160.95, 158.71, 152.63, 150.77, 148.50, 139.96, 133.62, 133.12, 132.68, 128.37, 128.22, 127.73, 126.97, 126.82, 126.61, 124.29, 123.41, 121.70, 111.86, 110.62, 109.34, 95.73, 92.41 (Ar–O–CH–), 71.02, 70.39, 68.58, 57.63, 56.32, 56.09, 55.90, 55.72, 53.87, 53.29, 47.75, 42.11. ESI Calcd for [M + H]^+^ C_38_H_40_N_2_O_9_^+^ 669.2807, found 669.2820.

#### 3–(2-(2–(4-(5-Bromo-2-chlorobenzoyl)piperazin-1-yl)ethoxy)ethoxy)-2–(3,4-dimethoxyphenyl)-5,7-dimethoxy-4H-chromen-4-one (11)

Yield:74%; yellow oil; ^1^H NMR (600 MHz, CDCl_3_) *δ* 7.76 (d, *J* = 1.4 Hz, 1H), 7.71 (dd, *J* = 8.5, 1.8 Hz, 1H), 7.42 (dd, *J* = 8.5, 2.3 Hz, 1H), 7.39 (d, *J* = 2.2 Hz, 1H), 7.24 (d, *J* = 8.5 Hz, 1H), 6.94 (d, *J* = 8.6 Hz, 1H), 6.49 (d, *J* = 2.0 Hz, 1H), 6.33 (d, *J* = 1.9 Hz, 1H), 4.25–4.21 (m, 2H), 3.94 (d, *J* = 7.9 Hz, 9H) (–OCH_3_)_3_, 3.89 (s, 3H) (–OCH_3_), 3.72 (dd, *J* = 10.6, 5.8 Hz, 4H), 3.53 (t, *J* = 5.6 Hz, 2H), 3.20 (dddd, *J* = 16.3, 10.3, 6.8, 3.0 Hz, 2H), 2.54–2.33 (m, 6H); ^13^C NMR (151 MHz, CDCl_3_) *δ* 173.87 (Ar–CO–), 165.05 (–CO–N), 163.89, 160.95, 158.72, 152.63, 150.76, 148.49, 139.95, 137.51, 133.08, 131.06, 130.62, 129.34, 123.40, 121.69, 120.85, 111.85, 110.61, 109.33, 95.74, 92.41 (Ar–O–CH–), 71.02, 70.38, 68.60, 57.53, 56.34, 56.09, 55.93, 55.74, 53.55, 52.94, 46.59, 41.59. ESI Calcd for [M + H]^+^ C_34_H_36_N_2_O_9_BrCl^+^ 731.1365, found 731.1387.

#### 3–(2-(2–(4-(Cyclopropanecarbonyl)piperazin-1-yl)ethoxy)ethoxy)-2–(3,4-dimethoxyphenyl)-5,7-dimethoxy-4H-chromen-4-one (12)

Yield: 67%; yellow oil; ^1^H NMR (600 MHz, CDCl_3_) *δ* 7.74 (d, *J* = 1.8 Hz, 1H), 7.71 (dd, *J* = 8.5, 1.9 Hz, 1H), 6.93 (d, *J* = 8.6 Hz, 1H), 6.48 (d, *J* = 2.1 Hz, 1H), 6.31 (d, *J* = 2.0 Hz, 1H), 4.23–4.19 (m, 2H), 3.93 (d, *J* = 10.6 Hz, 9H) (–OCH_3_)_3_, 3.87 (s, 3H) (–OCH_3_), 3.73–3.69 (m, 2H), 3.59 (d, *J* = 27.1 Hz, 4H), 3.52 (t, *J* = 5.7 Hz, 2H), 2.45 (dd, *J* = 36.9, 31.1 Hz, 6H), 1.70–1.64 (m, 1H) cyclopropyl, 0.95–0.91 (m, 2H) cyclopropyl, 0.73–0.69 (m, 2H) cyclopropyl; ^13^C NMR (151 MHz, CDCl_3_) *δ* 173.86 (Ar–CO–), 171.78 (–CO–N), 163.88, 160.94, 158.70, 152.65, 150.77, 148.49, 139.95, 123.39, 121.73, 111.85, 110.64, 109.31, 95.73, 92.41 (Ar–O–CH–), 71.03, 70.36, 68.59, 57.61, 56.31, 56.09, 55.90, 55.72, 53.78, 53.18, 45.29, 41.94, 10.83, 7.24 (2 C). ESI Calcd for [M + H]^+^ C_31_H_38_N_2_O_9_^+^ 583.265, found 583.2652.

### Cell culture

Cells were obtained from the Cell Resource Centre of Shanghai Institute for Biological Sciences. RAW264.7 and L-02 cells were cultured in DMEM (Hyclone, Logan, UT, USA) containing 10% FBS (Biological Industries, Beit-Haemek, Israel), 100 U/ml penicillin, and 100 μg/ml streptomycin (Beyotime, Jiangsu, China) at 37 °C in a hypoxic environment of 5% CO_2_.

### Release of NO assay

RAW264.7 cells were seeded in a 48-well plate at a density of 5 × 10^4^ cells per well. After incubated 24 h, compounds (20 μM) were added and 1 h later, 0.5 μg/ml LPS was added and incubated 24 h. Then 50 μl of supernatant was taken to detect the release of NO in a 96-well plate with Griess reagent (Beyotime, Jiangsu, China).

### Cell viability assay

MTT assay was used to evaluate the cell viability of compounds. RAW264.7 and L-02 cells were diluted to 5 × 10^4^ cells/ml^−1^ with the complete medium, and subsequently, 100 μL of the obtained cell suspension was added to each well of 96-well culture plates, were incubated at 37 °C, 5% CO_2_ atmosphere for 24 h. And then 100 μl drug-containing medium was dispensed into wells to maintain the final concentration as 40 μM. After 48 h exposure period, 20 μl PBS containing 5 mg/ml of MTT (3–(4,5-dimethylthiazol-2-yl)-2,5-diphenyltetrazolium bromide) (Sigma-Aldrich, St. Louis, MO) was added to each well. After 4 h of incubation at 37 °C, medium in 96-well plates was replaced by 150 μl DMSO (Sigma-Aldrich, St. Louis, MO, USA). The absorbance at 492 nm of each well was measured on an ELISA plate reader.

### Determination secretion of TNF-α and IL-6

RAW264.7 cells were seeded in a 48-well plate at a density of 5 × 10^4^ cells per well. After incubated 24 h, compound **8** (10, 5, and 2.5 μM) was added and 1 h later, 0.5 μg/ml LPS was added and incubated for 24 h. Cell supernatant was collected. The levels of TNF-α and IL-6 in the supernatant were measured by ELISA (eBioScience, San Diego, CA, USA).

### In vivo experiment

The male 18–22 g C57BL/6 mice were obtained from the Animal Department of Anhui Medical University. After one week of acclimatisation, mice were randomly allocated into four groups: control group, LPS group, compound **8** (15 mg/kg) + LPS group, and compound **8** (30 mg/kg) + LPS group (*N* = 10 for each group). After animals were received an equal volume of compound **8** by intraperitoneal injection for 30 min, inflammation was induced by tracheal instillation with 5 mg/kg LPS. Body weight was recorded. After 24 h, mice were anaesthetised and taken blood. The levels of IL-6 and TNF-α in serum were measured by ELISA (eBioScience, San Diego, CA, USA). The research was approved by the Ethics Committee of Anhui Medical University on the care and use of the Animal Centre, and all animals received humane care according to the National Institutes of Health (Bethesda, MD, USA) guidelines.
